# Development of a Nostalgic Remembering Intervention: Feeling Safe in Dyads Receiving Palliative Care for Advanced Heart Failure

**DOI:** 10.1097/JCN.0000000000000762

**Published:** 2020-11-12

**Authors:** Julie Fleury, Pauline Komnenich, David W. Coon, Barbara Volk-Craft

**Affiliations:** **Julie Fleury, PhD, FAAN, FAHA** Hanner Memorial Endowed Professor, Edson College of Nursing and Health Innovation, Arizona State University, Phoenix.; **Pauline Komnenich, PhD** Professor and Director MS in Nursing Program, Edson College of Nursing and Health Innovation, Arizona State University, Phoenix.; **David W. Coon, PhD** Associate Dean for R.I.S.E., Professor, and Director, Center for Innovation in Healthy and Resilient Aging, Edson College of Nursing and Health Innovation, Arizona State University, Phoenix.; **Barbara Volk-Craft, PhD** Special Projects Coordinator, Hospice of the Valley, Phoenix, AZ.

**Keywords:** geriatrics, heart failure, intervention development, palliative care

## Abstract

Dyads receiving palliative care for advanced heart failure are at risk for the loss of feeling safe, experienced as a fractured sense of coherence, discontinuity in sense of self and relationships, and strained social connections and altered roles. However, few theory-based interventions have addressed feeling safe in this vulnerable population.

**Purpose:**

The purpose of this article is to describe the development of the Nostalgic Remembering Intervention to strengthen feeling safe and promote adaptive physiological and psychological regulation in dyads receiving palliative care for heart failure.

**Conclusions:**

Systematic intervention development is essential to understand what, for whom, why, and how an intervention works in producing outcomes. Program theory provided a systematic approach to the development of the Nostalgic Remembering Intervention, including conceptualization of the problem targeted by the intervention, specification of critical inputs and conditions that operationalize the intervention, and understanding the mediating processes leading to expected outcomes.

**Clinical Implications:**

Creating a foundation for cardiovascular nursing research and practice requires continued, systematic development of theory-based interventions to best meet the needs of dyads receiving palliative care for heart failure. The development of the Nostalgic Remembering Intervention to strengthen feeling safe in dyads provides a novel and relevant approach.

Although medical therapies have reduced mortality rates due to cardiovascular disease, increasing numbers of older adults and their family caregivers now live with advanced heart failure (New York Heart Association functional class III–IV), along with multiple comorbid conditions.^1^ Heart failure is the most common diagnosis of hospitalized older adults, affecting approximately 8 million US adults, with an expected increase of 46% from 2012 to 2030.^2^ Palliative care in advanced heart failure provides an essential interdisciplinary resource, focused on improving quality of life and reducing suffering in care recipients and caregivers.^3^ Indeed, family caregivers are central to palliative care research and practice, as the relationship between the care recipient and caregiver determines the physical health and wellness of each.

Chronic illness such as advanced heart failure disrupts the integrity of the self and relationships. Older people with advanced heart failure and their caregivers experience fragmented coherence, discontinuity in sense of self and relationships, and disruption in social connections and family roles.^4^ The unpredictable course of advanced heart failure fosters uncertainty and fear, along with helplessness and powerlessness.^5,6^ As disease advances, older people express a loss of self in the illness role, with fear and worry that they have become a burden.^7^ Both caregivers and care recipients express negative changes in long-established roles and relationships.^8^ Furthermore, both experience social isolation, loneliness, and a sense of abandonment as social connection is diminished.^7^

Acknowledgment of the interdependence between older people with heart failure and family caregivers has informed dyadic approaches to research.^9^ For example, the quality of the caregiver-care recipient relationship may reduce caregiver burden and distress, allowing caregivers to better care for themselves and the care recipient.^7^ Among heart failure dyads, Nimmon and colleagues^8^ note the importance of living day to day as a unified “we,” reflecting reciprocity, care, and investment in each other. Kim and colleagues^10^ provide support for love and affection in the dyadic relationship as shaping the experience of heart failure management. Bangerter and colleagues^11^ explored the positive aspects of caring for a person with heart failure, finding that caregiving may enhance social ties and shift interpersonal relationships in positive ways. Mutuality in the dyadic relationship is associated with better outcomes related to self-care^7,12^ and anxiety and depression.^13^

Research exploring dyadic processes in heart failure supports the centrality of the dyadic relationship and feeling safe within the dyad. Feeling safe is more than the absence of threat; feeling safe relies on learned safety signals associated with protection from threat, found in familiar patterns and coherence, continuity in sense of self and relationships, and reliable close connection with others.^14,15^ Feeling safe is a distinct affective dimension characterized by warmth, affiliative connection, and calming, with increased parasympathetic nervous system activity and adaptive physiological and psychological regulation.^16–19^ Feeling safe is associated with higher vagally mediated heart rate variability (HRV), a measure of cardiovagal parasympathetic activity.^20^ In research examining the autonomic correlates of emotions, Duarte and Pinto-Gouveia^18^ found a significant quadratic relationship between parasympathetic vagal activity, measured by high-frequency HRV and safeness/soothing affect, but not activating a positive affect or a general measure of positive emotions. Similarly, Schwerdtfeger and Gerteis^21^ found that feeling safe, content, and calm, but not activating positive affect, was associated with higher nocturnal vagal tone (HRV). Increased vagal activity in feeling safe decreases the production of proinflammatory cytokines and may dampen systemic inflammation.^22^ Higher HRV also results in greater emotional well-being,^23^ self-regulation of emotion,^24^ improved social function, and feelings of connectedness.^25^

Feeling safe is associated with memories of home, of important people, and of experiences that are full of warmth and love. Memories from the past that engage safety signals are associated with feeling safe in the present, promoting adaptive physiological and psychological regulation.^26,27^ Memories of childhood warmth and safety have been linked to better health, fewer chronic illnesses, and fewer depressive symptoms in both middle and older adulthood.^28^ Consistent with these findings, we propose nostalgic remembering, “a sentimental longing for the past” as a novel intervention approach engaging safety signals found in familiar patterns and coherence, continuity in sense of self and relationships, and reliable close connections in dyads receiving palliative care for heart failure.^14,15^ Engaging safety signals found in the shared memories of coherence, continuity, and connection will strengthen feeling safe in the dyad and contribute to physiological and psychological regulation and improved heart failure and dyadic outcomes. Although nostalgic remembering has not been widely tested as an intervention in dyads receiving palliative care for heart failure, current literature supports the potential of this approach in a vulnerable population.^29,30^

Despite the acknowledged role of feeling safe in adaptive physiological and psychological regulation, few interventions have addressed feeling safe in the dyad receiving palliative care for heart failure.^26^ Dyadic intervention research in heart failure has primarily targeted disease management in the care recipient, including adherence to medical treatment, self-monitoring, and symptom support, with variable effectiveness.^31^ A systematic review of heart failure dyadic interventions evaluated current research as of low to moderate quality, noting poor articulation of dyadic intervention components, lack of theory-based critical content, and limited theory-based rationale for why and how a given intervention should work.^32^ Heart failure is characterized by autonomic imbalance, cardiovascular dysregulation, and inflammation; however, very few interventions have targeted autonomic function or evaluated physiological processes.^33^ Interventions rely primarily on cognitive processes, which may have limited explanatory value.^34^ Importantly, intervention research described as dyadic has varied in the degree to which the content and approach addressed the care recipient, the caregiver, or both. Most heart failure intervention research treats the care recipient and caregiver separately, limiting understanding of the interdependent nature of the dyad and missing an opportunity to strengthen feeling safe in the dyad.^3^

Theory-based dyadic interventions in advanced heart failure and palliative care are essential to expand the knowledge base for clinical practice, guiding cardiovascular nurses in implementing the most appropriate interventions. Walker and Czajkowski^35^ have advocated for rigorous intervention development before efficacy testing. Program theory provides a systematic approach to intervention development by specifying why, how, and under what conditions intervention effects occur, the proposed outcomes of the intervention, and the resources needed to realize intervention effects. The purpose of this article is to describe the development of the Nostalgic Remembering Intervention, which strengthens feeling safe and contributes to adaptive physiological and psychological regulation among dyads receiving palliative care for heart failure. Intervention development was guided by program theory as advanced by Lipsey^36^: (*a*) conceptualization of the problem targeted by the intervention, as well as specification of the population responsive to the intervention; (*b*) specification of the critical inputs and conditions that operationalize the intervention; (*c*) understanding of the mediating processes leading to expected outcomes; (*d*) identifying intervention implementation issues specific to materials and resources; and (*e*) identifying the exogenous factors that may influence intervention delivery.

## Elements of Nostalgic Remembering Intervention Development

### Problem Definition

An important function of theory in intervention development is to guide understanding of the nature and characteristics of the problem targeted by the intervention, as well as how the problem is experienced by the population of interest. Understanding the problem is critical for generating responsive intervention approaches, whereas failure to specify the nature of the problem limits the ability to tie the problem clearly to an intervention. Problem definition goes well beyond recognition of disease processes to include details of essential attributes for which intervention could provide a solution.^35^ Slippage between the problem and the intervention increases error, limits the effectiveness of the intervention in addressing the problem of interest, and reduces the ability to make valid causal inferences.^37^

The problem of interest is conceptualized as vulnerability for loss of feeling safe among dyads receiving palliative care for heart failure, experienced as a fractured sense of coherence, discontinuity in sense of self and relationships, and strained social connections and altered roles.^5,6^ In a systematic review of aspects of quality of life important to patients in palliative care, McCaffrey and colleagues^38^ found that the loss of feeling safe, as well as the need to feel safe, was pervasive across cognitive, emotional, healthcare, personal autonomy, physical, preparatory, social, and spiritual dimensions of quality of life. The loss of feeling safe is a primary determinant of autonomic nervous system imbalance associated with cardiovascular risk and functional decline.^14^ Autonomic imbalance with excessive sympathetic activity and reduced parasympathetic activity is indexed as lower vagal HRV. Lower HRV is associated with increased mortality among older people with heart failure,^39^ as well as increased hypothalamic-pituitary-adrenal (HPA) axis activation and chronic inflammation.^40^ Lower HRV has been linked with greater perceived difficulty in everyday emotion regulation,^41^ impaired social engagement, and decline in cognitive function.^42^

### Critical Inputs

Theory-driven interventions are developed in the context of the problem of interest, with critical inputs specifying what should be done, as well as what is necessary, sufficient, and optimal to prevent or manage the problem, thereby producing expected outcomes.^35^ Critical inputs clarify what constitutes treatment and what does not, thus stating the essential aspects of the intervention and the possibility for variation in intervention delivery, minimizing the possibility of alternative explanations for effects, and strengthening internal validity.^37^

Nostalgic remembering as an approach to intervention is distinct from autobiographical approaches such as life review and reminiscence. Life review and reminiscence facilitate problem solving and cognitive reappraisal of threats to self or resources, reflected in “big stories” of life events and disruptions.^43^ In contrast, nostalgia is experienced as autonoetic remembering, a positive self-referential reliving of the past.^44^ Rather than attempting to create a single and coherent life story, nostalgic remembering is linked to a specific person, place, or time with a positive association. Nostalgic remembering brings the past into the present, with the warmth, intimacy, and feeling of the familiar.^45^ In nostalgic remembering, people revisit moments shared with close others, reflected in “small stories” of comfort and belonging. Nostalgic remembering is experienced as positive and sweet,^46^ consistent with a calming physiological and emotional response.

In the Nostalgic Remembering Intervention, care recipients and caregivers recall and narrate a nostalgic memory from their shared past, engaging safety signals as critical inputs of familiar patterns and coherence, continuity in sense of self and relationships, and reliable close connections. Nostalgic remembering supports a sense of coherence, fostering predictability and reliable connections.^30^ Sense of coherence is an important determinate of wellness in caregivers and may lead to reduced psychological distress and perceived burden.^47^ As noted by May,^48^ our sense of self is forged out of memories. Nostalgic remembering among older adults promotes self-continuity,^37^ a perspective of the self, embedded in social relationships across time, and essential for positive function in later life. Research exploring the benefits of nostalgic remembering among older adults with mild to moderate levels of dementia found that nostalgia enhanced psychological resources and improved recall and recognition of self-referent information,^49^ consistent with self-continuity. Nostalgic remembering increases social connectedness, secure attachment, and social support, reinforcing reliable and close connections in the dyad.^48^

### Mediating Processes

Specifying mediating processes allows an understanding of how intervention critical inputs lead to proposed outcomes. The change process is contingent upon accurate specification and manipulation of mediating variables; interventions are more likely to be effective if the mediating variables are related to the intervention outcomes and if the intervention clearly targets change in the mediating variables.^37^

The Nostalgic Remembering Intervention strengthens feeling safe by engaging safety signals found in familiar patterns and coherence, continuity in sense of self and relationships, and reliable close connection with others.^14,26,50^ This contributes to adaptive physiological and psychological regulation and improved heart failure and dyadic outcomes (Figure).

**FIGURE:**
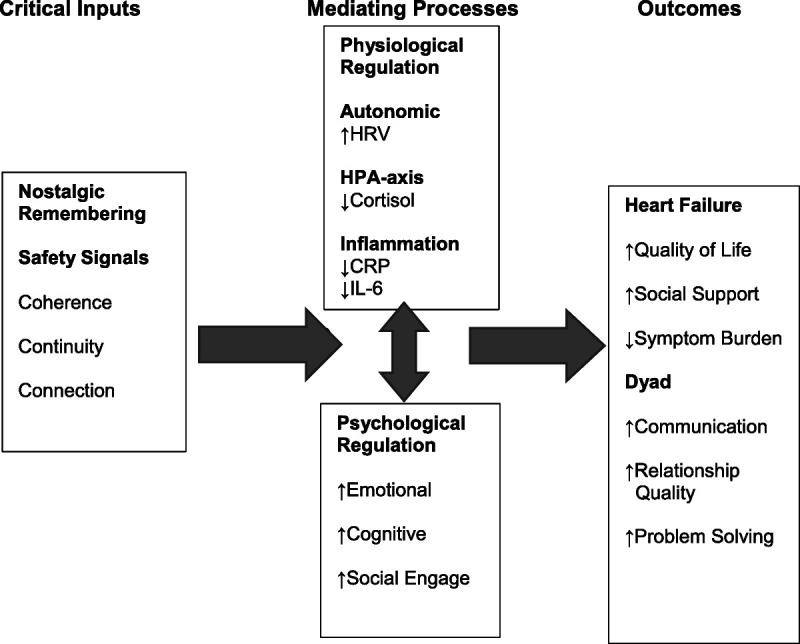
Nostalgic Remembering Intervention.

Dyads seek a “safe haven” in each other, engaging shared safety signals with inhibition of the amygdala through the input of the ventromedial prefrontal cortex and the hippocampus, increasing parasympathetic nervous system activity.^34,50^ The adaptive physiological response to safety signals reflects autonomic balance with increased HRV, decreased HPA axis activity, and reduced inflammation.^51,52^ Increased HRV is associated with lower cortisol levels, supporting a balance between the autonomic nervous system and the HPA axis.^53^ Reexperiencing connection with close supportive others, as in nostalgic remembering, contributes to increased HRV,^54^ decreased noradrenergic response,^55^ and decreased blood pressure reactivity.^56^ Matsunaga and colleagues^57,58^ found that odor-evoked nostalgic memories significantly decreased levels of peripheral proinflammatory cytokines.

Kemp and colleagues^59^ provide support for the bidirectional relationship between vagally mediated HRV and adaptive physiological and psychological response. Adaptive psychological response reflects positive affective processing of safety signals and the subjective feeling of security and comfort in response to parasympathetic activity.^60,61^ Mccall and Singer^19^ note that positive feelings of warmth and soothing are uniquely associated with parasympathetic activity. Higher HRV has been associated with feelings of soothing positive affect and social belonging,^18^ emotional well-being,^23^ and social connectedness.^25^ Feeling safe in nostalgic remembering is experienced as affective meaning,^62^ comfort and security,^63^ warmth,^64^ gentle contentment,^62^ and belonging,^65^ including feeling loved and protected.^46^

According to Porges,^26^ shared safety enables coregulation of parasympathetic nervous system activity in the dyad, beginning with the mother-infant bond and extending through the lifespan to other significant relationships. Social engagement fosters calm behavioral states by inhibiting sympathetic influence on the heart, dampening HPA axis activity and decreasing inflammation.^66^ The communication of shared safety relies on the bidirectional connection between the heart and the striated muscles of the face and head, with facial expression and voice inhibiting sympathetic influence on the heart.^67^ The Nostalgic Remembering Intervention in dyads leverages this face-heart connection, using the communication of shared safety to coregulate through reciprocal interaction, kind and engaging facial expression, softened eye contact, slowed breathing pattern, and prosodic voice, with both members feeling safe, calm, and bonded.^26^

### Expected Outcomes

Intervention outcomes reflect the prevention, resolution, or management of the problem targeted by the intervention. The outcomes selected are consistent with the target problem, relevant to the population of interest, and responsive to the intervention.^37^ By specifying the logic that connects intervention critical inputs to mediating and outcome variables, theory identifies the outcomes that can reasonably be expected.

The expected outcomes of the Nostalgic Remembering Intervention include those reflecting physiological and psychological self-regulation, as well as improved patient-centered heart failure and dyadic metrics.^68^ Autonomic function is evaluated as HRV, a primary outcome in intervention research exploring the efficacy of approaches promoting feeling safe and socially connected.^69^ HPA axis and immune response associated with HRV are evaluated as cortisol, C-reactive protein, and proinflammatory cytokines.^52^ Increased HRV, decreased HPA axis activity, and reduced inflammation may reduce symptom burden.^70^ Feeling safe fosters adaptive social, emotional, and cognitive function^26^ and is experienced as warmth, care, belonging, and soothing.^71^ Higher HRV is associated with greater emotional well-being^23^ and feelings of connectedness central to quality of life and perceived social support.^25^ Furthermore, outcomes of the Nostalgic Remembering Intervention in the dyad might include improved communication patterns, enhanced connection and relationship quality, and more effective problem solving around disease management.

### Exogenous Factors

Exogenous factors include contextual, environmental, or dyadic factors that may influence delivery of the Nostalgic Remembering Intervention. Fidelity in intervention delivery may be influenced by the type and quality of the relationship between the care recipient and the informal caregiver, including the ability and comfort of the dyad to engage in nostalgic remembering. For example, adult children serving as informal caregivers may have different nostalgic memories and may coregulate differently from a partner or spouse. Enactment of nostalgic remembering may be limited by chronic illness, multimorbidity, severe mental illness, or poor physical function, including difficulty in speaking, shortness of breath, fatigue, or extreme pain. Dyads participating in nostalgic remembering will be without significant cognitive impairment or noncorrectable hearing loss.

### Implementation Issues

Implementation issues include aspects of intervention delivery, including material resources and skill in facilitating the delivery of the intervention as planned. The Nostalgic Remembering Intervention will be delivered in a calm and quiet environment, with positive and warm interventionist interactions.^26^ Nostalgic Remembering Intervention sessions will take place in the home or a private location chosen by the dyad, to ensure comfort. Intervention delivery is evaluated specific to critical content and objectives, time spent in nostalgic remembering, and response to nostalgic remembering.

## Conclusions

Dyads receiving palliative care for heart failure experience the loss of feeling safe due to fragmented coherence, discontinuity in sense of self and relationships, and disruption in social connections and family roles.^4^ Despite the centrality of feeling safe to adaptive physiological and psychological regulation, few interventions have addressed feeling safe in the dyad.^26^ Feeling safe is a distinct affective dimension characterized by warmth, affiliative connection, and calming, with increased parasympathetic nervous system activity and inhibition of sympathetic nervous system activity.^18,27^ Feeling safe has a direct effect on autonomic function and related psychological and physiological regulation, rather than a buffering effect.^20^ Furthermore, intentionally strengthening feeling safe may provide a basis for voluntary upregulation of HRV and self-regulation of autonomic function.^72^

The Nostalgic Remembering Intervention in dyads receiving palliative care for heart failure provides a theory-based approach moving beyond a focus on disease management to advance feeling safe as an integrative and relational resource. The program theory facilitated a systematic approach to the development of the Nostalgic Remembering Intervention, specifying why, how, and under what conditions intervention effects occur, the proposed outcomes of the intervention, and the resources needed to realize intervention effects.^36^

Creating a foundation for theory-based intervention research requires that researchers and clinicians continue to identify areas for development relevant to feeling safe among dyads receiving palliative care for heart failure. Further research is required to better understand how the Nostalgic Remembering Intervention influences psychological and physiological regulation and coregulation. Issues such as the strength, dosage, and timing of the Nostalgic Remembering Intervention to realize effects remain to be considered. Longitudinal randomized controlled trials are needed to evaluate the sustainability of changes in autonomic nervous system and HPA-axis activity and immune function.^72^ A better understanding of potential moderating variables, including comorbid conditions such as hypertension and diabetes, will be important to research efforts.^59^ Perceived stress, sensitivity to social disconnection, anxiety and depression symptoms, and sleep disturbance may also serve as relevant moderators.^73^ Acknowledging that inflammation is both a cause and an outcome of heart failure and disease progression, bidirectional relationships in inflammatory mechanisms of change remain to be explored.^74^

As noted by Brosschot and colleagues,^14^ approaches which strengthen feeling safe engage and build upon sources of safety rather than promoting cognitive or emotional coping addressing the loss of feeling safe. Similarly, Levine^75^ advocates for intervention approaches that move beyond a narrow focus on managing cardiovascular diseases to those addressing the social, cognitive, emotional, and overall physical well-being of those we care for. In the Nostalgic Remembering Intervention, dyads receiving palliative care for heart failure navigate the future by reflecting on the past; finding safety in continuity, coherence, and enduring social connections in a life experience that may feel fragmented and unpredictable. Although nostalgic remembering has not been widely tested as an intervention approach in dyads receiving palliative care for heart failure, the use of program theory in development of the Nostalgic Remembering Intervention supports the potential of this approach.^29,30^

**What’s New and Important**

- Research evaluating dyadic interventions in heart failure has grown; however, the use of theory guiding the development of these interventions is limited. Theory-based interventions in advanced heart failure and palliative care are essential to develop and expand the knowledge base for clinical practice, guiding cardiovascular nurses in implementing the most appropriate interventions.
- Program theory provided a systematic approach to theory-based development of the Nostalgic Remembering Intervention, including conceptualization of the problem targeted by the intervention, specification of critical inputs and conditions that operationalize the intervention, and understanding the mediating processes leading to expected outcomes.
- The Nostalgic Remembering Intervention in dyads receiving palliative care for heart failure moves beyond a focus on heart failure disease management to advance feeling safe as an integrative and relational resource. Nostalgic remembering provides a novel approach, intentionally engaging signals of safety to strengthen feeling safe and adaptive physiological and psychological regulation.
